# Impact of Polo-like kinase 1 inhibitors on human adipose tissue-derived mesenchymal stem cells

**DOI:** 10.18632/oncotarget.12482

**Published:** 2016-10-05

**Authors:** Andreas Ritter, Alexandra Friemel, Nina-Naomi Kreis, Frank Louwen, Juping Yuan

**Affiliations:** ^1^ Department of Gynecology and Obstetrics, School of Medicine, J. W. Goethe-University, Theodor-Stern-Kai 7, D-60590 Frankfurt, Germany

**Keywords:** adipose tissue-derived stem cell, Plk1 inhibitors, mitotic arrest, apoptosis, senescence

## Abstract

Polo-like kinase 1 (Plk1) has been established as one of the most promising targets for molecular anticancer intervention. In fact, various Plk1 inhibitors have been identified and characterized. While the data derived from the bench are prospective, the clinical outcomes are less encouraging by showing modest efficacy. One of the explanations for this discrepancy could be unintendedly targeting of non-malignant cells by Plk1 inhibitors. In this work, we have addressed the effect of Plk1 inhibition in adipose tissue-derived mesenchymal stem cells (ASCs). We show that both visceral and subcutaneous ASCs display monopolar spindles, reduced viability and strong apoptosis induction upon treatment with BI 2536 and BI 6727, the Plk1 kinase domain inhibitors, and with Poloxin, the regulatory Polo-box domain inhibitor. While Poloxin triggers quickly apoptosis, BI 2536 and BI 6727 result in mitotic arrest in ASCs. Importantly, survived ASCs exhibit DNA damage and a pronounced senescent phenotype. In addition, Plk1 inhibition impairs ASCs' motility and homing ability. These results show that Plk1 inhibitors target slowly proliferating ASCs, an important population of anti-inflammation and immune modulation. The toxic effects on primary cells like ASCs could be partially responsible for the reported moderate antitumor activity in patients treated with Plk1 inhibitors.

## INTRODUCTION

The Polo-like kinase (Plk) family is a group of highly conserved serine/threonine kinases. Plk1, the best studied member of this family, is a key regulator of multiple stages of mitosis including centrosome maturation, spindle formation, chromosome segregation and cytokinesis [1]. Moreover, Plk1 is highly expressed in various entities of malignancy and is closely correlated with poor prognosis of tumor patients [2]. Plk1 has been thus regarded as one of the most promising targets for molecular anticancer therapy [2, 3]. It contains two functional domains, the N-terminal kinase domain and the C-terminal regulatory Polo-box domain (PBD), which offer multiple targeting strategies by blocking the ATP-binding pocket of its kinase domain, like BI 2536 [4] and BI 6727 (volasertib) [5, 6], or inhibiting the function of the unique PBD, such as Poloxin [7, 8]. In addition, there is a type II inhibitor SBE13, targeting the inactive kinase domain of Plk1 [9–12]. The effect of Plk1 inhibition in tumor cells is well characterized: it induces monopolar spindles, mitotic arrest and apoptosis, leading further to reduced proliferation *in vitro* and inhibited tumor growth *in vivo* [2]. While the data derived from cancer cell lines are promising, the clinical results are less encouraging by displaying moderate efficacy associated with side-effects including neutropenia, leukopenia, and thrombocytopenia [3, 13–15]. One of the molecular mechanisms for the dissatisfaction could be ascribed to unintendedly targeting of non-malignant cells by Plk1 inhibitors.

In fact, numerous investigations were performed to understand the potential effect of Plk1 inhibition in primary/normal cells like fibroblasts, mammary epithelial cells and human umbilical vein endothelial cells (HUVEC) [8, 16–18]. These studies have reported that, like in tumor cells, Plk1 inhibitors work efficiently in various primary/normal non-transformed dividing cells with only a slightly less sensitivity [8, 17, 18]. In particular, Plk1 inhibition profoundly impacts primary cardiac fibroblasts by arresting them in mitosis followed by cell death or aneuploidy [16], suggestive of a concern of Plk1 inhibitors by targeting non-malignant proliferating cells.

Mesenchymal stem cells (MSCs) are known for their differentiation capability into multiple cell types such as osteoblasts, adipocytes, and chondrocytes [19, 20]. They reside in diverse adult tissues, such as adipose tissue, being referred to as adipose tissue-derived mesenchymal stem cells (ASCs) [21], bone marrow [20], lung [22] and heart [23]. MSCs are capable of responding to microenvironmental signals and recruit themselves toward the places where they are required, like inflammatory and wounded sites [24], and play crucial roles in tissue repair, anti-inflammation, angiogenesis and immune modulation [25, 26]. In the present work, we have addressed if and how Plk1 inhibition impacts human ASCs. Therefore, we isolated these cells from subcutaneous and visceral adipose tissues and analyzed their cellular phenotype, mitotic distribution, proliferation rate, motility behaviour, and apoptosis induction upon treatment with distinct Plk1 inhibitors to clarify the potential cytotoxicity of Plk1 inhibition in slowly proliferating mesenchymal stem cells.

## RESULTS

### ASCs from visceral and subcutaneous adipose tissue are susceptible to small molecule inhibitors targeting Plk1

ASCs proliferate in a slow rate compared to tumor cells [27]. To address if ASCs respond to Plk1 inhibitors, we isolated ASCs from visceral and subcutaneous adipose tissues from female donors undergoing caesarean sections as reported [27, 28]. The clinical information of patients is listed in Table 1. To determine the purity of these cells, we examined the typical cell surface marker profile for mesenchymal stem cells described by the Society of Cellular Therapy [28, 29]. The data from flow cytometry showed that both ASCs, namely visceral ASCs and subcutaneous ASCs, were highly positive for CD90, CD73, CD146, CD105 and negative for CD14, CD31, CD106 and CD34 (Table 2), characteristic for ASCs [29].

**Table 1:** Clinical information of 15 patients

	age	gestational age (weeks)	body mass index (BMI)	birth weight (g)
patients	32.2 ±3.1	39.1 ± 2.4	23.2 ± 2.2	3187 ± 651

**Figure 1:**
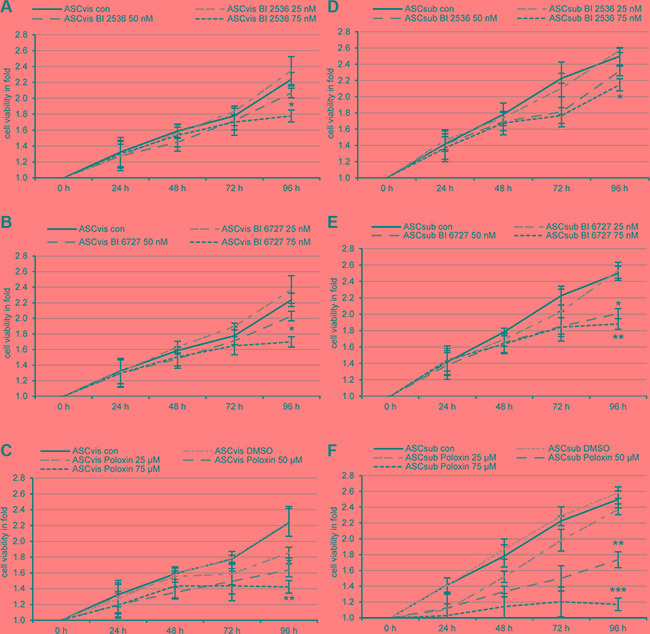
Small molecule inhibitors against Plk1 reduce the cell viability of visceral and subcutaneous ASCs Both subtypes of ASCs were seeded in 96-well plates and treated with indicated concentrations of BI 2536 (**A**, **D**), BI 6727 (**B**, **E**) or Poloxin (**C**, **F**) for 0, 24, 48, 72 and 96 h. Cell viability was measured via CellTiter-Blue^®^ assay. DMSO treated cells served as vehicle control. The results are from three independent experiments, presented as mean ± SEM and statistically analyzed compared to DMSO treated cells. *p < 0.05, **p < 0.01, ***p < 0.001.

Both ASCs were treated with Plk1 inhibitors BI 2536 and BI 6727 targeting the kinase domain [4, 5] and Poloxin against the PBD [8] for 0, 24, 48, 72 and 96 h at concentrations used for tumor cells as reported previously [30, 31], and cellular viability was evaluated. Visceral ASCs expanded slightly slower (Figure 1A–1C) than subcutaneous ASCs (Figure 1D–1F). Despite this difference, both ASCs were prone to all three Plk1 inhibitors by showing a significant proliferative inhibition after the treatment (Figure 1). The inhibitory response in ASCs took place at 72 and 96 h upon the treatment, in contrast tumor cells showed reduced proliferation already at 48 to 72 h [27]. This difference could be ascribed to their different doubling time and the generally fast growth rate of tumor cells. Visceral ASCs responded more sensitively to BI 2536 at 75 nM (Figure 1A and 1D), whereas subcutaneous ASCs were more susceptible to BI 6727 at 50 nM (Figure 1B and 1E). Intriguingly, both ASCs, in particular, subcutaneous ASCs, were sensitive to Poloxin, the PBD inhibitor (Figure 1C and 1F). These results demonstrate that ASCs, one type of MSCs, are well targeted by small molecule inhibitors against Plk1 by showing proliferative inhibition.

### Mitotic arrest of ASCs upon the treatment with Plk1 inhibitors

**Figure 2:**
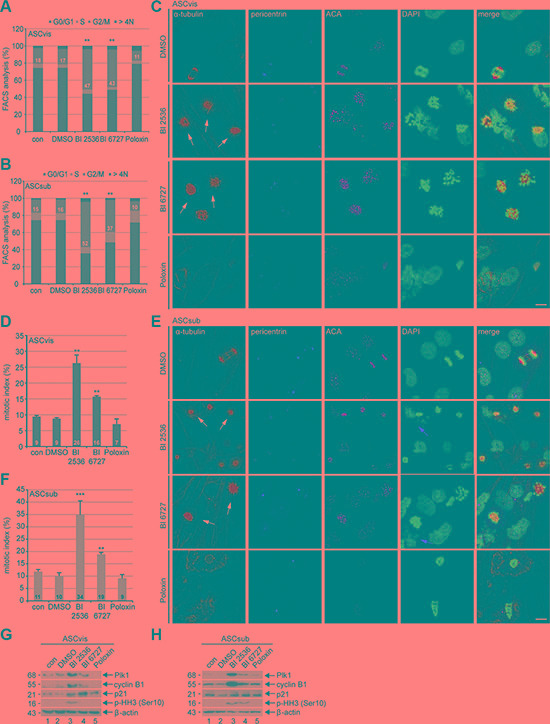
Plk1 inhibition triggers mitotic arrest in ASCs (**A** and **B**) ASCs were treated with indicated Plk1 inhibitors (50 nM BI 2536, BI 6727 or 50 μM Poloxin) for 48 h and cell cycle analyses were performed for visceral and subcutaneous ASCs. The results are based on three independent experiments, presented as mean ± SEM and statistically analyzed compared to DMSO treated cells. **p < 0.01. (C and E) ASCs were treated as in (A and B) and stained for α-tubulin, pericentrin, ACA (anti-centromere antibody) and DNA for immunofluorescence microscopy. Mitotic arrested ASCs were evaluated. Representatives are shown for visceral ASCs (**C**) and subcutaneous ASCs (**E**). White arrows indicate mitotic cells; red arrows indicate fragmented cell nuclei. Scale: 25 μm. (**D** and **F**) Quantification of mitotic cells (*n* = 150 analyzed cells for each condition). The results are from three independent experiments and presented as mean ± SEM. **p < 0.01, ***p < 0.001. (**G** and **H**) Cellular extracts from treated ASCs were prepared for Western blot analyses with indicated antibodies. β-actin served as loading and DMSO treated cells as vehicle control.

To further investigate the reduced cellular viability, we studied the cell cycle profile of treated cells. ASCs were treated with 50 nM BI 2536, 50 nM BI 6727 and 50 μM Poloxin for 48 h and harvested for flow cytometry. While BI 2536 and BI 6727 induced an increased G2/M peak of 21–36% in both subtypes of ASCs, Poloxin reduced even the G2/M population compared to control cells (Figure 2A and 2B). Treated ASCs were also stained for microtubule marker α-tubulin, the centrosome marker pericentrin, the centromere/kinetochore marker ACA (anti-centromere antibody) and DNA for immunofluorescence microscopy. Compared to non-treated control and DMSO treated cells, visceral ASCs displayed 17% and 8% more prometaphase cells upon BI 2536 and BI 6727 treatment, respectively (Figure 2C, 1^st^ to 3^rd^ panel and 2D). Poloxin treated visceral ASCs showed an increase in apoptotic cells, with fragmented DNA, diffuse ACA and α-tubulin staining, leading to a slight decrease of mitotic cells (Figure 2C, 4^th^ panel and 2D). Subcutaneous ASCs responded in a similar way, cells treated with BI 2536 exhibited an increase in mitotic cells of 23%, whereas BI 6727 led to an increase of 8% relative to control cells (Figure 2E, 1^st^ to 3^rd^ panel, and 2F). Treatment with Poloxin stimulated again DNA fragmentation (Figure 2E, last panel). Western blot analysis further corroborated a mitotic arrest evidenced by increased mitotic proteins Plk1, cyclin B1 and phospho-histone H3 (p-HH3, Ser10) in both BI compound treated ASCs, in particular BI 2536, compared to control cells (Figure 2G and 2H, 1^st^, 2^nd^ and 4^th^ row). In addition, relative to control cells, the expression of the stress protein p21^Cip1/CDKN1A^ (p21) was enhanced in both treated ASCs, especially in visceral ASCs (Figure 2G and 2H, 3^rd^ row). Moreover, visceral ASCs treated with Poloxin displayed hardly signals in any of the three mitotic markers, which could be due to the induction of apoptosis (Figure 2G, 5^th^ row).

### Monopolar spindles in ASCs induced by Plk1 inhibition

**Figure 3:**
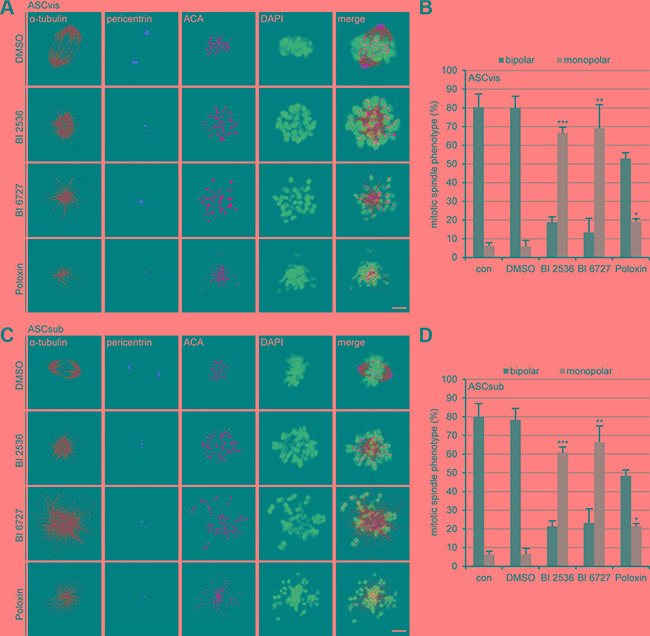
The inhibition of Plk1 induces monopolar spindles in both ASC subtypes (**A**) Visceral ASCs were treated with 25 nM BI 2536, 25 nM BI 6727 or 25 μM Poloxin for 24 h. Cells were stained with indicated antibodies and representative confocal images are presented. DMSO treated cells served as vehicle control. Scale: 5 μm. (**B**) The spindle form of mitotic visceral ASCs (*n* = 100 mitotic cells for each condition) was evaluated and defined as monopolar and bipolar spindles. The results are from three independent experiments, presented as mean ± SEM and statistically analyzed, compared to DMSO treated cells. *p < 0.05, **p < 0.01, ***p < 0.001. (**C**) Subcutaneous ASCs were treated as in (A). ASCs were then stained with indicated antibodies and representative confocal images are shown. DMSO treated cells served as vehicle control. Scale: 5 μm. (**D**) The spindle form of mitotic subcutaneous ASCs (*n* = 100 mitotic cells for each condition) was evaluated and classified as monopolar and bipolar spindles. The results are from three independent experiments, presented as mean ± SEM and statistically analyzed, relative to DMSO treated cells.*p < 0.05, **p < 0.01, ***p < 0.001.

Next we were interested in the mitotic phenotype induced by Plk1 inhibition. To get enough cells for this analysis, ASCs were treated for 24 h with a lower concentration of the compounds: 25 nM BI 2536, 25 nM BI 6727 or 25 μM Poloxin. Treated cells were stained for α-tubulin, pericentrin, ACA and DNA for immunofluorescence microscopy. BI 2536 and BI 6727 generated significantly more monopolar spindles relative to DMSO treated cells (Figure 3A–1D, 1^st^ to 3^rd^ panels in A and C). Whereas Poloxin increased moderately the rate of monopolar spindles (Figure 3A–3D, 4^th^ panels in A and C), it induced also aberrant bipolar spindles (data not shown), similar to treated tumor cells, as previously described [31]. Remarkably, both ASC subtypes showed barely difference upon Plk1 suppression regarding the percentages of induced monopolar spindles (Figure 3B and 3D).

**Figure 4:**
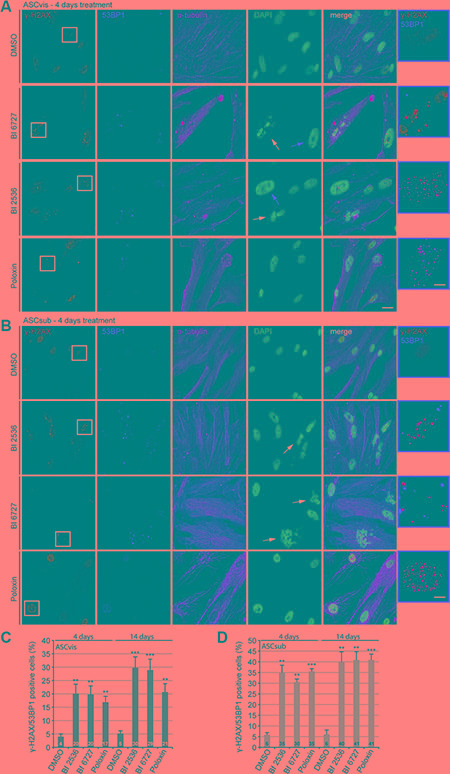
Plk1 inhibitor treatment induces apoptosis in both subtypes of ASCs ASCs were subjected to Plk1 compounds (25 nM BI 2536, 25 nM BI 6727 or 25 μM Poloxin) for 4 days and 14 days. Media and compounds were renewed every 3 days. **(A** and **B)** To evaluate the induction of DNA damage, treated cells were stained for the DNA damage markers γ-H2AX and 53BP1, α-tubulin and DNA. Representatives are shown. White arrows indicate fragmented cell nuclei and red arrows depict abnormal enlarged cell nuclei. Scale: 25 μm. Insets are a four time magnification of boxed regions in leftist panels. Scale: 6.5 μm. (**C** and **D**) Quantification of γ-H2AX and 53BP1 double positive cells. The results are based on three independent experiments and presented as mean ± SEM. **p < 0.01, ***p < 0.001.

### Prolonged Plk1 inhibition induces DNA damage and apoptosis in ASCs

As Plk1 is involved in DNA damage response by regulating important kinases like Chk1 and Chk2, which are responsible for DNA damage sense and repair [32], we were then interested if the survived cells after prolonged Plk1 inhibition display DNA damage. ASCs were subjected to low concentrations of the compounds for 4 days, followed by the staining of DNA damage markers γ-H2AX (Ser139) and p53-binding protein 1 (53BP1), α-tubulin and DNA for microscopic analysis. Increased γ-H2AX/53BP1 foci, characteristic of DNA strand breaks, were easily observed in survived cells (Figure 4A and 4B). Further evaluation showed that all three compounds increased significantly the number of γ-H2AX/53BP1 double positive cells in both ASC subtypes, in particular, in subcutaneous ASCs (Figure 4C and 4D, 4 days). To study if ASCs exhibit durable DNA damage under Plk1 inhibition, ASCs were subjected to the Plk1 inhibitors for 14 days followed by the staining. Under this condition, the percentage of double positive cells was further elevated in both ASC subtypes, especially again in subcutaneous ASCs (Figure 4C and 4D, 14 days). Interestingly, both BI 2536 and BI 6727 induced cells with highly enlarged nuclei in visceral ASCs (Figure 4A, 2^nd^ and 3^rd^ panel, red arrows) suggesting that polyploidy/aneuploidy could take place in those treated ASCs, as reported for cardiac fibroblasts [16]. Furthermore, either ASCs treated with BI 2536 or BI 6727 showed DNA fragmentation (Figure 4A and 4B, 2^nd^ and 3^rd^ panel, withe arrows), suggestive of apoptosis induction.

**Figure 5:**
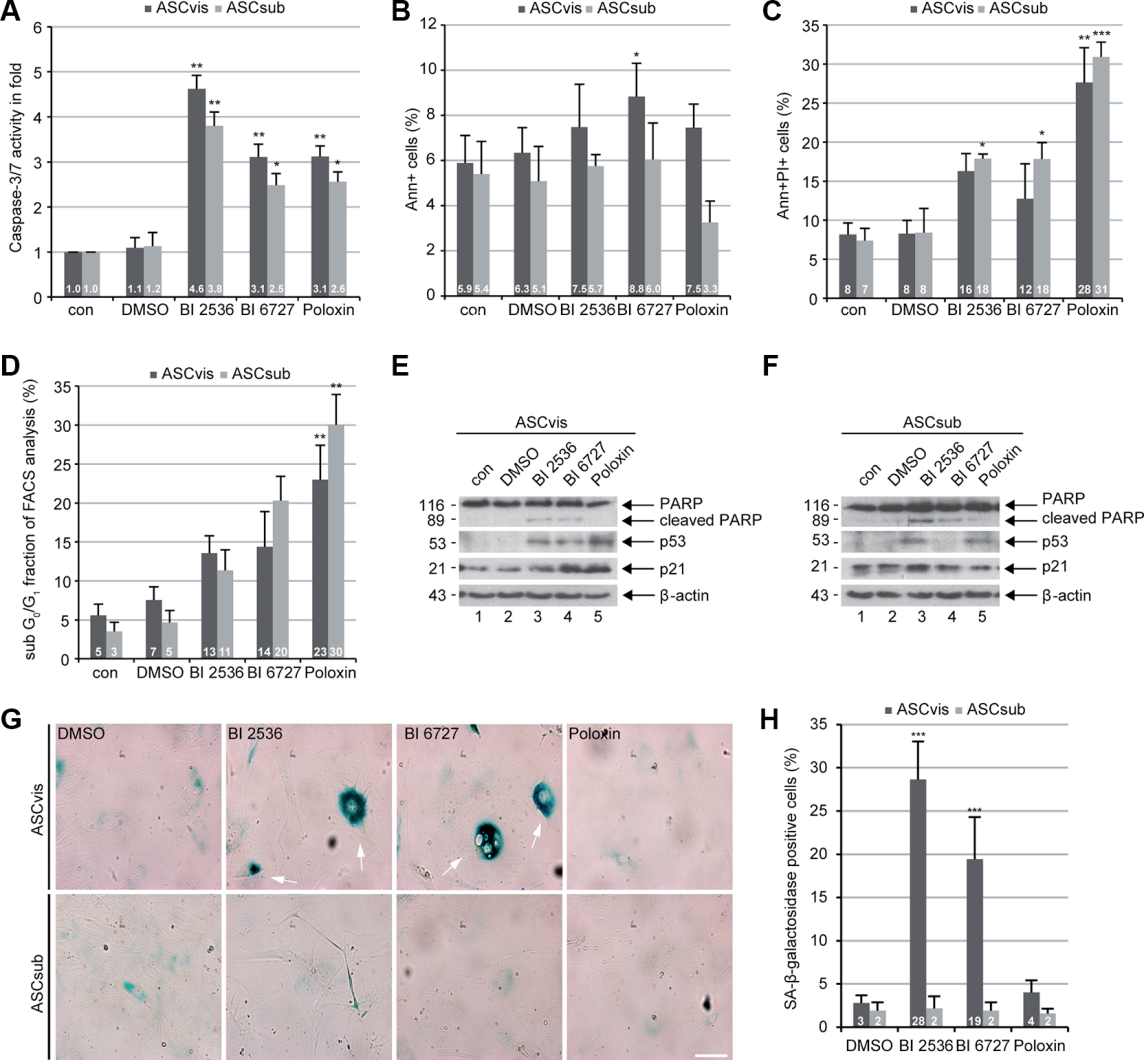
Plk1 inhibition induces apoptosis in both ASCs and senescence in survived visceral ASCs (**A**) ASCs were treated with Plk1 compounds (50 nM BI 2536, 50 nM BI 6727 or 50 μM Poloxin) for 96 h. The relative activity of caspase-3/7 was evaluated in treated ASCs. The results are based on three independent experiments and shown as mean ± SEM. *p < 0.05, **p < 0.01. (**B** and **C**) Treated ASCs were harvested for early and late apoptosis measurements via flow cytometry. Annexin V positive (Ann+, B), annexin V and PI double positive cells (Ann+PI+, C) were quantified. The results were obtained from three independent experiments and displayed as mean ± SEM. DMSO treated cells served as vehicle control. *p < 0.05, **p < 0.01 ***p < 0.001. (**D**) Treated cells were also subjected to cell cycle measurements via flow cytometry. The sub G_0_/G_1_ fraction was evaluated. The results are from three independent experiments and shown as mean ± SEM. **p < 0.01. (**E** and **F**) Cellular extracts from treated ASCs were prepared for Western blot analyses with indicated antibodies. β-actin served as loading and DMSO treated cells as vehicle control, respectively. (**G**) ASCs were treated with DMSO, 50 nM BI 2536, 50 nM BI 6727 or 50 μM Poloxin for 8 days followed by staining of senescence-associated β-galactosidase (SA-β-gal; blue-green). Scale: 50 μm. (**H**) Evaluation of SA-β-gal positive cells (at least *n* = 100 cells for each condition, except in Poloxin treated ASCs: *n* = 50). The results are based on three independent experiments, presented as mean ± SEM and statistically analyzed. ***p < 0.001.

Apoptosis induction was further demonstrated by the evaluation of caspase-3/7 activity (Figure 5A), Annexin V positive staining (Figure 5B), Annexin V/propidium iodide (PI) double positive staining (Figure 5C) and sub G_0_/G_1_ peaks in flow cytometric analysis (Figure 5D) of ASCs treated with Plk1 inhibitors for 96 h. Notably, while BI 2536 provoked an induction of early apoptosis (Figure 5A), Poloxin triggered a stronger late apoptotic response in ASCs (Figure 5C and 5D). Moreover, Western blot analysis underscored the apoptosis induction. The enhanced cleavage of poly (ADP-ribose) polymerase (PARP), an apoptosis marker, was observed in both ASCs treated with BI 2536 and BI 6727 (Figure 5E and 5F, 1^st^ row). In short, the results demonstrate a significant toxic potency of Plk1 compounds in slowly dividing ASCs. In addition, Plk1 inhibition elevated the level of the tumor suppressor p53 in both subcutaneous and visceral ASCs (Figure 5E and 5F, 2^nd^ row), in line with the notion that Plk1 negatively regulates the stability of p53 by downstream targets like GTSE1 or Topors [3, 33, 34]. Notably, while subcutaneous ASCs showed a generally high level of p21 (Figure 5F, 3^rd^ row), visceral ASCs displayed an enhanced p21 after Plk1 inhibition for 96 h (Figure 5E, 3^rd^ row), in accordance with the observation in ASCs after 24 h treatment (Figure 2G, 3^rd^ row).

### Long-term treatment with Plk1 inhibitors induces senescence in visceral ASCs

Next, we investigated if survived ASCs are able to escape the cell cycle arrest and go into cellular senescence, as previously reported for cancer cells [35]. To address this question, an established senescence-associated β-galactosidase assay (SA-β-gal) [36] was carried out in both ASCs treated for up to eight days with Plk1 inhibitors. Strikingly, visceral ASCs were easily switched into the senescence state, being flattened, enlarged, multinucleated and SA-β-gal-positive, upon BI compound treatment in a highly significant extent compared to DMSO treated control cells (Figure 5G and 5H). This phenomenon was neither observed in subcutaneous ASCs nor in Poloxin treated visceral ASCs (Figure 5G and 5H).

### The inhibition of Plk1 impairs the motility of ASCs

**Figure 6:**
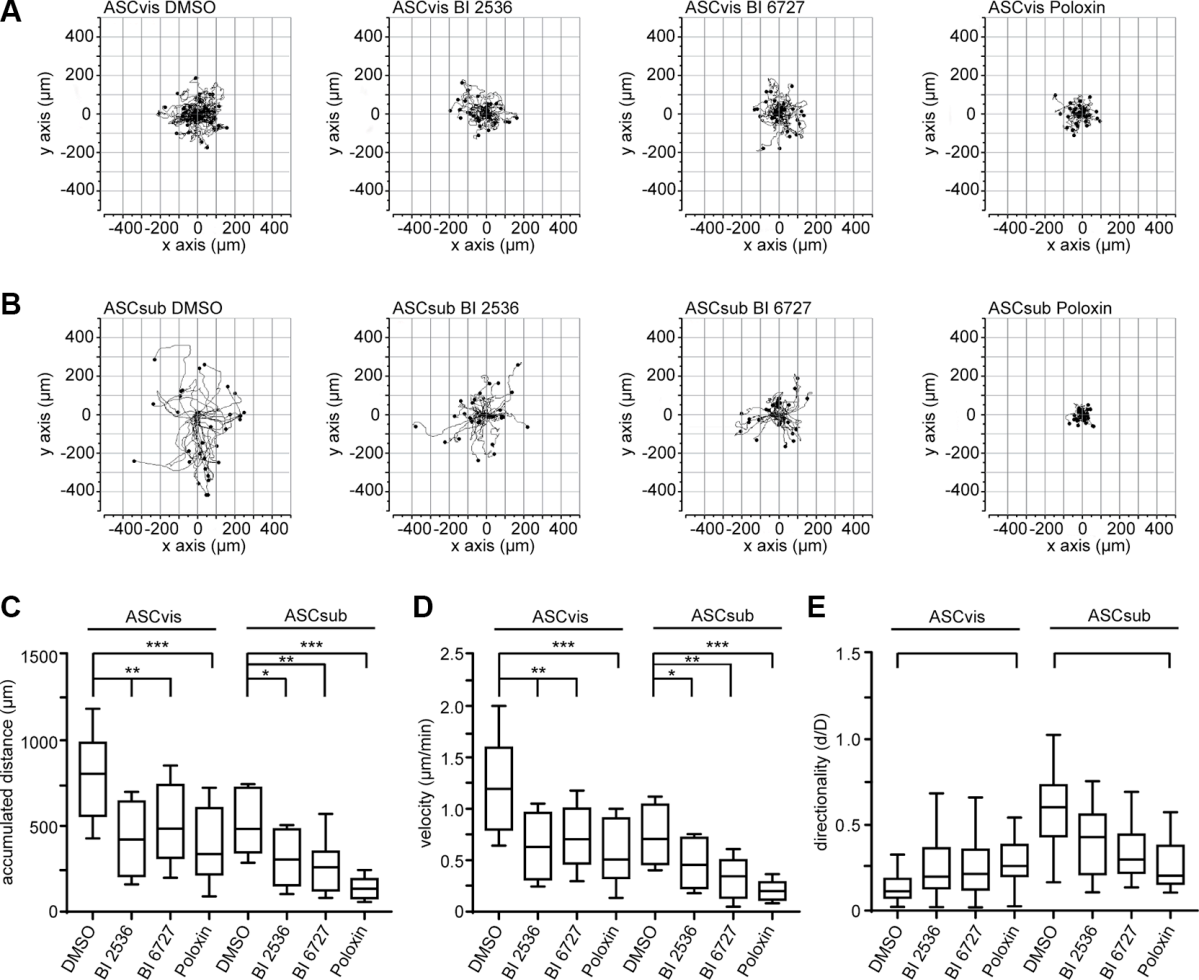
The inhibition of Plk1 reduces the random motility of ASCs ASCs were treated with BI 2536 (25 nM), BI 6727 (25 nM) or Poloxin (25 μM) up to 12 h and released into fresh medium. Cells were then subjected to time-lapse microscopy for analyzing their random motility. (**A** and **B**) Representative trajectories of individual treated cells (*n* = 30 cells). Measurement of the accumulated distance (**C**), the evaluation of the velocity (**D**) and the directionality of cell migration (**E**) are presented. The results are from three independent experiments, shown as mean ± SEM and statistical analyzed by an unpaired Mann-Whitney test. *p < 0.05, **p < 0.01, ***p < 0.001.

The Plk1 expression level is well known to be tightly correlated with the invasion of tumor cells [2]. Interestingly, it has been recently revealed that Plk1 promotes the motility of epithelial cells by activating CRAF/ERK signaling [37]. Mesenchymal stem cells are highly motile [38]. To define the motility of ASCs upon Plk1 suppression, time-lapse microscopy was performed as reported [39, 40]. We tracked individual single ASCs treated with BI 2536, BI 6727 and Poloxin up to 12 h and measured the random movement of individual cells as reported [37]. To exclude motility alterations related to cytoskeleton changes in mitotic arrested cells induced by Plk1 inhibition, only interphase cells were analyzed during the whole evaluation time frame. Interestingly, the accumulated migratory distance and the velocity of both ASC subtypes were significantly decreased after treatment with any of the three Plk1 compounds compared to control cells (Figure 6A–6D). Both ASCs exhibited similar effects: in comparison to control cells, visceral ASCs had a reduced velocity of 39.8–59.1% (Figure 6D) and subcutaneous ASCs displayed a reduction of 38.1–63.3% depending on the used compound (Figure 6D). Surprisingly, ASCs derived from subcutaneous and visceral adipose tissues have different migration patterns: subcutaneous ASCs showed an intrinsic directionality, whereas visceral ASCs migrated in a non-directed fashion (Figure 6E). Moreover, subcutaneous ASCs showed a highly significant loss of directionality upon treatment with Plk1 inhibitors (Figure 6E). By contrast, visceral ASCs demonstrated a moderate increase in directionality after treatment with Plk1 inhibitors (Figure 6E). These results strongly suggest that Plk1 is required for an accurate migratory behavior of ASCs.

**Figure 7:**
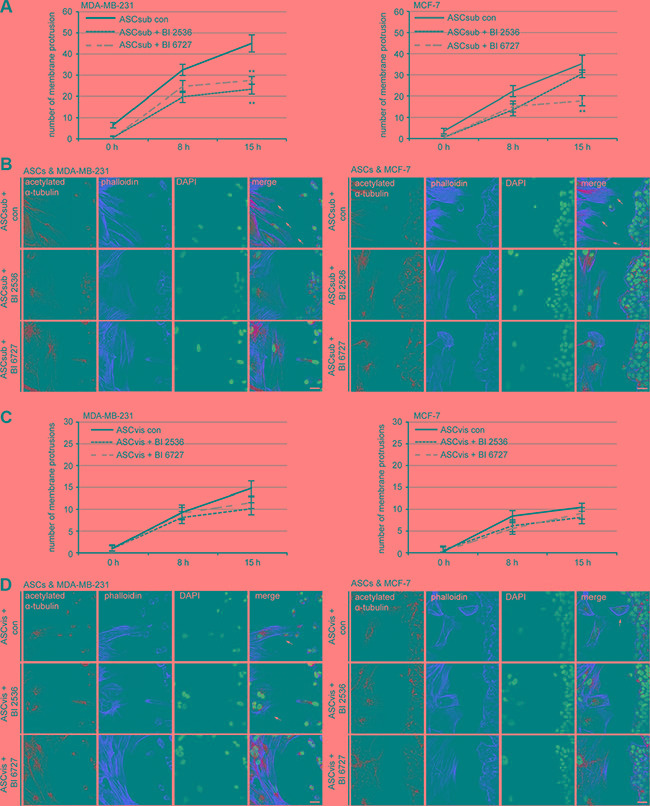
Plk1 inhibition reduces the homing ability of subcutaneous ASCs ASCs were treated with Plk1 inhibitors (50 nM BI 2536 or 50 nM BI 6727) for 9 h and released into fresh media. Treated ASCs and breast cancer cells were seeded in separated chambers of an culture insert and cultured for 0 h, 8 h and 15 h. Cells were then stained for microtubule marker acetylated α-tubulin, actin marker phalloidin and DNA for analyzing the number of membrane protrusions, including lamellipodia and filopodia, on the migration front of ASCs toward breast cancer cells. (**A**) Evaluation of protrusions in subcutaneous ASCs toward MDA-MB-231 and MCF-7 cells. Each experiment was performed in triplicate, and the results are from three independent experiments and shown as mean ± SEM. **p < 0.01. (**B**) Representatives are depicted. Left side: subcutaneous ASCs; right side: breast cancer cells. Scale: 25 μm. (**C**) Evaluation of protrusions in visceral ASCs toward MDA-MB-231 and MCF-7 cells. The results are from three independent experiments and shown as mean ± SEM. (**D**) Representatives are shown. Left side: visceral ASCs; right side: breast cancer cells. Scale: 25 μm.

To study the impact of Plk1 inhibition on the homing ability of ASCs toward injured or inflammatory sites [38], we performed an established attraction assay with two different breast cancer cell lines [27]. ASCs were treated with Plk1 inhibitors for a short time (9 h) and released into fresh media to avoid a hindered homing ability related to apoptosis induction. Treated ASCs and breast cancer cells were then seeded in separated chambers of a culture insert with a defined cell free gap between these two chambers. The number of membrane protrusions including lamellipodia and filopodia of ASCs toward cancer cells was evaluated in the time frame between 0 h to 15 h evidencing the tropism of ASCs. The homing ability of subcutaneous ASCs toward the breast cancer cell lines MCF-7 and MDA-MB-231 showed a significant decrease even after the short time treatment with Plk1 inhibitors (Figure 7A and 7B), supporting the results of the impaired migration behavior obtained by the single cell tracking assay (Figure 6A–6E). Intriguingly, the attraction rate of visceral ASCs is just moderately decreased upon inhibitor treatment (Figure 7C and 7D), presumably due to their preferred non-directional migration mode. In conclusion, the motility of both ASC subtypes is strongly inhibited by Plk1 inhibitors, which influence direct cell-cell interactions as shown in the attraction assay.

## DISCUSSION

As Plk1 targets multitudinous substrates throughout the cell cycle and beyond, a complete understanding of Plk1 inhibition is not yet achieved. Plk1 is a key regulator essential for all dividing cells, regardless of malignant or normal cells. To define undesired effects and the long-term outcome of its usage, it is of utmost importance to understand the impact of Plk1 inhibitors on various primary cells. In the present work, we have systematically studied the effects of different Plk1 inhibitors on ASCs. We show that both visceral and subcutaneous ASCs exhibit a reduced cell viability accompanied by a strong apoptosis induction after treatment with the Plk1 kinase domain inhibitors BI 2536 and BI 6727 as well as with the PBD inhibitor Poloxin. Treated ASCs display monopolar spindles, characteristic of Plk1 inhibition [30, 31]. While Poloxin triggers quickly apoptosis, BI 2536 and BI 6727 result in a mitotic arrest as a first response in treated ASCs. Importantly, Plk1 inhibition induces DNA damage in both subtypes of ASCs, in particular, in subcutaneous ASCs. A senescent phenotype is clearly observed in treated visceral ASCs. Moreover, Plk1 inhibition impedes ASCs' motility and homing ability, which are pivotal for their functionalities.

ASCs are multipotent cells, which are capable of differentiation into a variety of cell types of different origins [41]. Responding to diverse microenvironmental cues, ASCs are capable of tissue repair/regeneration, angiogenesis, anti-inflammation and immune modulation [26]. Deregulated/impaired ASCs could be detrimental for disease progression. Indeed, obesity induces alterations in the biologic properties of ASCs, subsequently leading to enhanced tumorigenesis and metastasis of cancer cells [42]. Functional competent ASCs are therefore crucial for preventing and combating malignancy by modulating and safeguarding a regulated “healthy” stromal environment. In addition, ASCs are considered as tools for anticancer gene therapy, based on both their tumor homing feature and the utility of ectopic gene expression [43]. On the other hand, recent publications report the involvement of ASCs in cancer development as well as cancer recurrence, indicating the impact of cancer cells on ASCs and a sophisticated interaction between cancer cells and their microenvironment [44, 45] and highlighting the importance of maintaining this cell population intact and competent against cancer cells. In this context, systematic administration of Plk1 inhibitors could reduce dramatically ASCs viability and impair their functionality by inducing apoptosis and altering their biological features like anti-inflammation, which are pivotal for the fate of tumor cells.

Senescent cells are known for their highly metabolic activity with diverse functions and context-dependent tissue reactions [46]. Senescence results from two steps, a cell cycle arrest by Cdk inhibition induced for example by p21 or p16 and geroconversion, an irreversible step driven by growth-signaling pathways such as mammalian target of rapamycin signaling (mTOR) [47]. Intriguingly, it has been suggested that there is a molecular and functional link between Plk1 and the mTOR pathway [48]. Indeed, the mTOR signaling regulates the catalytic activity of Plk1 via Mio, an activator for mTORC1 [49], whereas Plk1 phosphorylates Rictor, a core component of mTORC2 [50]. We show here that both BI compounds induce senescence in visceral ASCs, in line with our previous observation that colon carcinoma HCT116 cells were senescent after a prolonged treatment with Plk1 inhibitors [30]. Moreover, it has been reported that partial inhibition of the Plk1 activity by using chemical genetics induced cellular senescence in human retinal pigment epithelial cells [51]. In further support of this notion, Plk1 depletion with small interfering RNA resulted in senescence in human dermal fibroblasts and human umbilical vein endothelial cells [52]. The activity of Plk1 is thus oppositely correlated to the induction of senescence in tumor as well as primary cells, mainly due to its important roles in the p53/p21 pathway [3, 30, 35, 53] and the possible link to with mTOR [48–50]. Since senescence could promote tumor development and contribute to therapy resistance [46, 54], the senescence induction upon Plk1 inhibition in tumor and normal cells could contribute to the moderate efficacy observed in patients treated with Plk1 inhibitors.

Furthermore, we show that suppression of Plk1 results in a compromised migratory behavior in both ASC subtypes, explained by Plk1's function in promoting cell motility by activating CRAF/ERK signaling [37]. This hampered migration, combined with the impaired cell viability, might affect the wound healing capacity of treated patients, as MSCs are fundamental for this process [38, 55]. Moreover, we show that survived ASCs contain DNA damage, in agreement with the observation that Plk1 is involved in DNA damage response by affecting the important regulators like Chk1 and Chk2 [32]. In addition, it is well established that Plk1 is involved in the DNA synthesis during the S phase under stress situation [56, 57], Plk1 inhibition could thus generate DNA damage in ASCs. These damaged ASCs could be more susceptible to become tumor associated ASCs.

In summary, these results demonstrate a toxic effect of all tested Plk1 inhibitors in primary visceral and subcutaneous ASCs. Plk1 is one of the most promising targets combating tumors. However, these observations from ASCs imply that, along with the inhibited tumor cells, systematic administration of Plk1 inhibitors could simultaneously deteriorate the functionalities of various primary dividing cells. In support of this assumption, the toxic effect of Plk1 inhibitors has been repeatedly reported in various normal primary proliferating cells, like cardiac fibroblasts and endothelial cells [8, 16]. Unintended toxic effects on primary cells, such as ASCs, could be partly responsible for the reported moderate anti-tumor activity in patients treated systematically with Plk1 inhibitors. Interestingly, the combination of chemotherapeutics with other drugs like metformin, rapamycin or actinomycin D protects primary cells from mitotic arrest [58–60]. Selectively targeted Plk1 inhibition and combined therapy with other agents should be the favored strategy for improving the efficacy and reducing undesired effects.

## MATERIALS AND METHODS

### Cell culture, human ASC isolation and inhibitors

Ethics approval was obtained from the Ethics Committee of the Johann-Wolfgang-Goethe University Hospital Frankfurt and informed written consent was obtained from all donors. MCF-7 and MDA-MB-231 were obtained from ATCC (Germany, Wesel). All cells were cultured as instructed. BI 2536 and BI 6727 were purchased from Selleck Chemicals LLC (Houston) and Poloxin was kindly provided by Dr. Berg (Leipzig University). DMSO was from Sigma- Aldrich (Taufkirchen).

Visceral (omental) and subcutaneous (abdominal) adipose tissues were taken from women undergoing caesarean section (Table 1). ASCs were isolated as described previously [28] and cultured under standard cell culture conditions. After 24 h, non-adherent cells were removed and the remaining cells were washed, cultured and expanded. Early passages (P2 to P5) of isolated ASCs were used for experiments and characterized for their cell surface marker profile (Table 2).

**Table 2:** Cell surface markers of ASCs

	Cell surface markers (in %)							
	CD90	CD73	CD146	CD105	CD14	CD31	CD106	CD34
ASCvis	78.78	73.07	41.02	92.32	4.99	1.99	2.54	1.41
± SD	12.91	15.49	18.64	2.26	5.31	0.76	2.10	1.87
ASCsub	79.75	87.39	50.49	96.75	6.39	2.06	2.07	1.97
± SD	11.93	11.69	16.30	0.88	6.67	0.76	1.57	1.11

Mean values of the percentage of specific surface markers on ASC (n = 10 patient samples). ASCvis, visceral adipose tissue-derived stem cells; ASCsub, subcutaneous adipose tissue-derived stem cells; SD, standard deviation.

### Cell proliferation assays, cell cycle analysis and apoptosis assays

Cell proliferation assays were performed by using Cell Titer-Blue^®^ Cell Viability Assay (Promega, Mannheim) as described [30]. Both ASCs were seeded with 3000 cells per 96-well plate and the cell proliferation was measured up to 96 h. Plk1 interfering compounds (BI 2536, BI 6727 and Poloxin) were added in indicated concentrations directly after seeding. The cell cycle distribution was analyzed using a FACSCalibur^TM^ (BD Biosciences, Heidelberg), as described [61, 62]. Briefly, cells were harvested, washed with PBS, fixed in chilled 70% ethanol at 4°C for 30 min, treated with 1 mg/ml of RNase A (Sigma-Aldrich) and stained with 100 μg/ml of propidium iodide (PI) for 30 min at 37°C. DNA content was determined.

Late apoptosis (Ann+PI+) were assessed using Vybrant™ apoptosis assay kit #2 according to the instructions (Molecular Probes, Leiden) [30]. Apoptosis was measured with a FACSCalibur^TM^ (BD Biosciences). The data were analyzed by using the cell cycle analysis software CellQuest (BD Biosciences). The activity of caspase-3/7 was measured in triplicate with Caspase-Glo^®^ 3/7 Assay as instructed (Promega).

### Cellular extract preparation and Western blot analysis

Cellular lysates were prepared using RIPA buffer (50 mM Tris pH 8.0, 150 mM NaCl, 1% NP-40, 0.5% Na-desoxycholate, 0.1% SDS, 1 mM NaF, 1 mM DTT, phosphatase and protease inhibitor cocktail tablets (Roche, Mannheim)). Western blot analysis was performed as previously described [53, 63]. Following antibodies were used: Mouse monoclonal antibodies against cyclin B1, Plk1 and p53 (Santa Cruz). Rabbit monoclonal antibodies against p21 and rabbit polyclonal antibodies against PARP were purchased from Cell Signaling. Anti-phospho-histone H3 (Ser10) were obtained from Merck Millipore. Mouse monoclonal antibody against β-actin was from Sigma-Aldrich.

### Immunofluorescence staining and microscopy

For immunofluorescence staining cells were fixed for 8–10 min with methanol at −20°C or with 4% paraformaldehyde containing 0.2% Triton X-100 for 15 min at room temperature as described previously [64]. The following primary antibodies were used: rat polyclonal antibody against α-tubulin (Biozol, Eching), rabbit polyclonal antibodies against pericentrin, human monoclonal antibody against ACA (anti-centromere antibody) (ImmunoVision, Springdale), mouse monoclonal anti-phospho-histone γ-H2AX (Ser139) (Merck Millipore, Darmstadt) and polyclonal rabbit antibodies against 53BP1 (Novus, Cambridge, UK). FITC-, Cy3- and Cy5 conjugated secondary antibodies were obtained from Jackson Immunoresearch. DNA was stained using DAPI (40,6-diamidino-2-phenylindole-dihydrochloride) (Roche, Mannheim). The actin cytoskeleton was stained using phalloidin (Phalloidin-Atto 550) (Sigma-Aldrich, Taufkirchen). Slides were examined using an AxioObserver.Z1 microscope (Zeiss, Göttingen) and images were taken using an AxioCam MRm camera (Zeiss). The immunofluorescence stained slides were further examined by confocal laser scanning microscopy (CLSM) using Z-stack images with a HCXPI APO CS 63.0 × 1.4 oil objective (Leica CTR 6500, Heidelberg) in sequential excitation of fluorophores. A series of Z-stack images were captured at 0.5 μm intervals. All images in each experiment were taken with the same wave intensity and exposure time. All experiments, unless otherwise indicated, were independently performed at least three times. Representatives shown in figures are generated by superimposing (overlay) individual images from confocal Z-sections.

### Senescence-associated β-galactosidase (SA-β-gal) assay

Senescence-associated β-galactosidase (SA-β-gal) assay was performed at pH 5.7 as instructed (Cell Signaling). Briefly, visceral and subcutaneous ASCs were seeded in chamber slides (Nunc^TM^ Lab-Tek^TM^ II CC2^TM^), treated up to 8 days, washed, fixed and stained with X-gal staining solution for 6 h at 37°C without CO_2_. The cells were observed with an AxioObserver.Z1 microscope (Zeiss), imaged with an AxioCam MRc camera (Zeiss) and the β-galactosidase positive cells were evaluated.

### Cell motility and attraction assay

Cells were seeded into 24-well plates with a low confluency and were imaged for 12 hours at 5 minute time intervals. All time-lapse imaging was performed with an AxioObserver.Z1 microscope (Zeiss), imaged with an AxioCam MRc camera (Zeiss) equipped with an environmental chamber to maintain proper environmental conditions (37°C, 5% CO_2_). The time-lapse movies were analyzed by using ImageJ 1.49i software (National Institutes of Health, USA) with the manual tracking plugin, and Chemotaxis and Migration Tool (Ibidi GmBH, Germany). Tracks were derived from raw data points and were plotted in GraphPad Prism 7 (GraphPad software Inc., USA). The accumulated distance was calculated by using the raw data points by the Chemotaxis and Migration Tool. Thirty random cells per experiment were analyzed and the experiments were repeated independently three times.

The patterns of motility were evaluated as descripted previously [37]. The migration velocity was calculated as the mean sum of all accumulated distances divided by the migration time (equation. 1). The unit is given in micrometers per minutes.

Equation 1 $$\mathrm{Migration}\;\mathrm{velocity}=\frac{1}{n}\sum_{i=1}^{n}\frac{di,accum}{t}$$

The directionality is measured by comparing the euclidian distance (d_i,euclid_) to the accumulated distance (d_i,accum_). Therefore, it represents a calculation of the directness of the cell trajectories (equation 2). The directness is a parameter to characterize the straightness of migration. The value of the directionality is given as a ratio between the euclidian distance and the accumulated distance simplified as [d/D].

Equation 2 $$\mathrm{Directionality}=\frac{1}{n}\sum_{i=1}^{n}\frac{di,euclid}{di,accum}$$

Cell attraction assays were performed with culture-inserts from ibidi (Martinsried). Culture-inserts (cell free gap of 500 μm) were placed in a 6-cm culture dish and one well of each insert was filled with cell suspensions either with Plk1 inhibitor treated cells (50 nM BI 2536 or BI 6727) visceral or subcutaneous ASCs (5.5 × 10^4^) or with the investigated cells (MCF-7: 5.5 × 10^4^, MDA-MB-231: 6.5 × 10^4^). After 9 h, the culture inserts were removed and the images were obtained at indicated time points. Cellular movement toward the other migration front was evaluated using the AxioVision SE64 Re. 4.9 software (Zeiss), for each experiment at least five migration front images were taken, analyzed and the experiments were performed in triplicate.

### Statistical analysis

Student's *t*-test (two tailed and paired or homoscedastic) was used to evaluate the significance of difference between different groups. The statistical evaluation of the single cell tracking assay was performed by using an unpaired Mann-Whitney test (two tailed). Difference was considered as statistically significant when *p* < 0.05.

## Abbreviations

ASC: adipose tissue-derived mesenchymal stem cell
MSC: mesenchymal stem cell
MT: microtubule
Plk: Polo-like kinase
PBD: polo-box domain
ACA: anti-centromere antibody.
